# Influence of intermittent fasting on myocardial infarction-induced cardiac remodeling

**DOI:** 10.1186/s12872-019-1113-4

**Published:** 2019-05-28

**Authors:** K. Okoshi, M. D. M. Cezar, M. A. M. Polin, J. R. Paladino, P. F. Martinez, S. A. Oliveira, A. R. R. Lima, R. L. Damatto, S. A. R. Paiva, L. A. M. Zornoff, M. P. Okoshi

**Affiliations:** 1 Internal Medicine Department, Botucatu Medical School, Sao Paulo State University, UNESP, Botucatu, SP Brazil; 2 Departamento de Clinica Medica, Faculdade de Medicina de Botucatu, UNESP, Rubiao Junior, S/N. CEP 18618-687, Botucatu, SP Brazil; 3 Itapeva Social and Agrarian Sciences College, FAIT, Itapeva, SP Brazil; 4 Federal University of Mato Grosso do Sul, Cidade Universitária, Av. Costa e Silva – Pioneiros, Campo Grande, MS 79070-900 Brazil

**Keywords:** Myocardial infarction, Intermittent feeding, Langendorff preparation, Fetal gene expression, Echocardiogram, Ventricular remodeling, Heart failure, Rat, Isolated heart, Calorie restriction

## Abstract

**Background:**

Information on the role of intermittent fasting (IF) on pathologic cardiac remodeling is scarce. We compared the effects of IF before and after myocardial infarction (MI) on rat cardiac remodeling and survival.

**Methods:**

Wistar rats were intermittently fasted (food available every other day) or fed ad libitum for 12 weeks and then divided into three groups: AL – fed ad libitum; AL/IF - fed AL before MI and IF after MI; and IF – fed IF before and after MI. Echocardiogram was performed before MI and 2 and 12 weeks after surgery. Isolated hearts were evaluated in Langendorff preparations.

**Results:**

Before surgery, body weight (BW) was lower in IF than AL. Final BW was lower in AL/IF and IF than AL. Perioperative mortality did not change between AL (31.3%) and IF (27.3%). Total mortality was lower in IF than AL. Before surgery, echocardiographic parameters did not differ between groups. Two weeks after surgery, MI size did not differ between groups. Twelve weeks after MI, left ventricular (LV) diastolic posterior wall thickness was lower in AL/IF and IF than AL. The percentage of variation of echocardiographic parameters between twelve and two weeks showed that MI size decreased in all groups and the reduction was higher in IF than AL/IF. In Langendorff preparations, LV volume at zero end-diastolic pressure (V0; AL: 0.41 ± 0.05; AL/IF: 0.34 ± 0.06; IF: 0.28 ± 0.05 mL) and at 25 mmHg end-diastolic pressure (V25; AL: 0.61 ± 0.05; AL/IF: 0.54 ± 0.07; IF: 0.44 ± 0.06 mL) was lower in AL/IF and IF than AL and V25 was lower in IF than AL/IF. V0/BW ratio was lower in IF than AL and LV weight/V0 ratio was higher in IF than AL. Myocyte diameter was lower in AL/IF and IF than AL (AL: 17.3 ± 1.70; AL/IF: 15.1 ± 2.21; IF: 13.4 ± 1.49 μm). Myocardial hydroxyproline concentration and gene expression of ANP, Serca 2a, and α- and β-myosin heavy chain did not differ between groups.

**Conclusion:**

Intermittent fasting initiated before or after MI reduces myocyte hypertrophy and LV dilation. Myocardial fibrosis and fetal gene expression are not modulated by feeding regimens. Benefit is more evident when intermittent fasting is initiated before rather than after MI.

## Background

Overweight and obesity prevalence has increased over recent decades in many parts of the world [1] and obesity has been associated with several comorbidities and increased mortality [2]. Many dietary regimens are used to decrease body weight and preserve a healthy body mass. Daily caloric restriction is probably the most common form of dietary restriction [3]. More recently, intermittent fasting has become an approach to energy restriction in humans [4]. Although observational data on the relationship between long-term intermittent fasting and risk of cardiometabolic disease are limited, there is evidence that both alternate-day fasting and periodic fasting may be effective for weight loss [3, 5]. Beneficial effects on cardiovascular risk factors have been reported including reductions in body fat, and total cholesterol and triglyceride levels [3–5].

Intermittent fasting has been evaluated in experimental animals under different scenarios. Usually mice and rats are deprived of food every other day and fed ad libitum on the intervening days [6]. Even in the absence of expressive body weight loss, intermittent fasting has been associated with lifespan extension, improved glucose regulation [7, 8], neuroprotection for memory loss [9–11], nephroprotection [6], and increased cellular resistance to various type of stress [12].

Information regarding the role of intermittent fasting on pathologic cardiac remodeling is scarce. Rats subjected to intermittent fasting both before and after myocardial infarction have demonstrated attenuation in both cardiac remodeling and left ventricular dysfunction [13, 14]. However, whether the beneficial effects of intermittent fasting initiated before and after myocardial infarction are similar is still unclear. In this study, we compared the effects of intermittent fasting before and after myocardial infarction on rat cardiac remodeling and survival.

## Methods

### Experimental groups

Two month-old male Wistar rats were acquired from the Central Animal House, Botucatu Medical School, UNESP. Animals were housed in a room under controlled temperature and light/dark cycle. Experimental design was approved by the Animal Experimentation Ethics Committee of Botucatu Medical School, UNESP, SP, Brazil.

The rats were randomly distributed to be fed either every day (ad libitum*,* AL) or every other day (intermittent fasting, IF) with a standard rat diet. After 12 weeks of these feeding regimens, all rats were subjected to coronary artery ligation and the following three groups were created: fed ad libitum (AL); fed ad libitum before MI and intermittently fasted after MI (AL/IF); and intermittently fasted before and after MI (IF). Cardiac structures and left ventricular (LV) function were assessed by transthoracic echocardiograms before MI induction, and 2 and 12 weeks after surgery. At the end of the experiment, after anesthesia, hearts were removed and mounted in the Langendorff apparatus (AL, *n* = 8; AL/IF, *n* = 9; IF, *n* = 13). All remaining animals were anesthetized with pentobarbital (50 mg/kg, intraperitoneal) and euthanized by thoracotomy and hearts removal. Right and left ventricles were dissected, weighed, frozen in liquid nitrogen, and kept at − 80 °C for molecular analyzes. Since in vitro perfusion can alter myocardium tissue, hearts used in Langendorff preparations were not subjected to additional evaluation.

### Myocardial infarction

After anesthesia with xylazine (10 mg/kg) and ketamine (70 mg/kg), MI was induced as described in our laboratory [15, 16].

### Echocardiographic study

Rats were lightly anesthetized with an intraperitoneal injection of ketamine (50 mg/kg) and xylazine (1 mg/kg). Echocardiogram was performed by the same blinded examiner (KO) using a echocardiograph (General Electric Medical Systems, Vivid S6, Tirat Carmel, Israel) equipped with a 5.0–11.5 MHz multifrequency probe, according to the previously described method [17–20]. MI size was estimated by two-dimensional image measuring end-diastolic endocardial perimeter of affected myocardium in relation to the total LV endocardial perimeter.

### Isolated LV study - Langendorff preparation

One day after echocardiographic evaluation, rats were anesthetized with sodium thiopental (50 mg/kg, intraperitoneal) and heparin (2000 IU, intraperitoneal) and euthanized by thoracotomy. After removing hearts, they were mounted in the Langendorff apparatus according to the method previously described [21–23]. After recording functional data, hearts were detached, the atria and great vessels removed, and the ventricles separated and weighed [23].

### Morphological analysis

Hematoxylin and eosin-stained slides from LV tissue were used to measure approximately 50 cardiomyocyte diameters as the lower distance between myocyte borders drawn across the nucleus [24–26].

### Myocardial hydroxyproline

Left ventricular concentration of hydroxyproline was quantified to estimate myocardial collagen content according to a previously described method [27–29]. In brief, myocardial samples were dried and hydrolyzed overnight at 100 °C with 6 N HCl (1 mL/10 mg dry tissue). Aliquots of 50 μL were dried again in a Speedvac Concentrator. After adding 1.0 mL of deionized water and 1.0 mL of potassium borate buffer (pH 8.7), samples were oxidized with 0.3 mL of chloramine T solution for 20 min. The addition of 1 mL of 3.6 M sodium thiosulfate stopped the oxidative process. The solution was then saturated with 1.5 g KCl. The tubes were heated in boiling water for 20 min. After extracting the aqueous layer with 2.5 mL of toluene, 1.5 mL of toluene extract were added to 0.6 mL of Ehrlich’s reagent and the color was allowed to develop for 30 min. Absorbances were read at 565 nm against a reagent blank. Deionized water and hydroxyproline standard (20 μg/mL) were used as the blank and control, respectively.

### Gene expression

To assess the fetal gene program, we analyzed sarcoplasmic reticulum calcium ATPase (Serca 2a), myocardial α- and β-myosin heavy chain, and atrial natriuretic peptide (ANP) expression by real time RT-PCR as previously described [30–33]. Briefly, total RNA was extracted, solubilized in RNase-free H_2_O, incubated in DNase I (Invitrogen Life Technologies), quantified and reverse transcribed. Aliquots of cDNA were submitted to real-time PCR using 10 μL 2X TaqMan® Universal PCR Master Mix (Applied Biosystems) and 1 μL of customized assay (20X) containing sense and antisense primers and Taqman (Applied Biosystems, Foster City, CA, EUA) probe specific to the following genes: Serca2a (Taqman assay Rn00568762; Ref. seq. Genbank NM_017290), α-myosin (myosin heavy polypeptide 6, cardiac muscle, alpha; Taqman assay Rn00568304_m1; Ref. seq. Genbank NM_017239.1), β-myosin (myosin heavy polypeptide 7, cardiac muscle, beta; Taqman assay Rn00568328_m1; Ref. seq. Genbank NM_017240.1), and natriuretic peptide precursor type A (Taqman assay Rn00561661_m1; Ref. seq. Genbank NM_012612.1). Amplification and analysis were then performed; reactions were carried out in triplicate. After normalizing data expression to cyclophilin (Taqman assay Rn00690933_m1; Ref. seq. Genbank NM_017101), results were calculated using the CT method (2^-ΔΔCT^).

### Statistical analyzes

Data are expressed as mean ± standard deviation or median and 25th and 75th percentiles in accordance with normal or non-normal distribution. Comparisons between groups were performed by analysis of variance (ANOVA) complemented by the Tukey’s test or Kruskal-Wallis and Dunn’s tests for normal and non-normal distributions, respectively. Mortality was assessed by log-rank test (Kaplan Meier). Statistical significance was accepted at the level of *p* < 0.05.

## Results

**Fig. 1:**
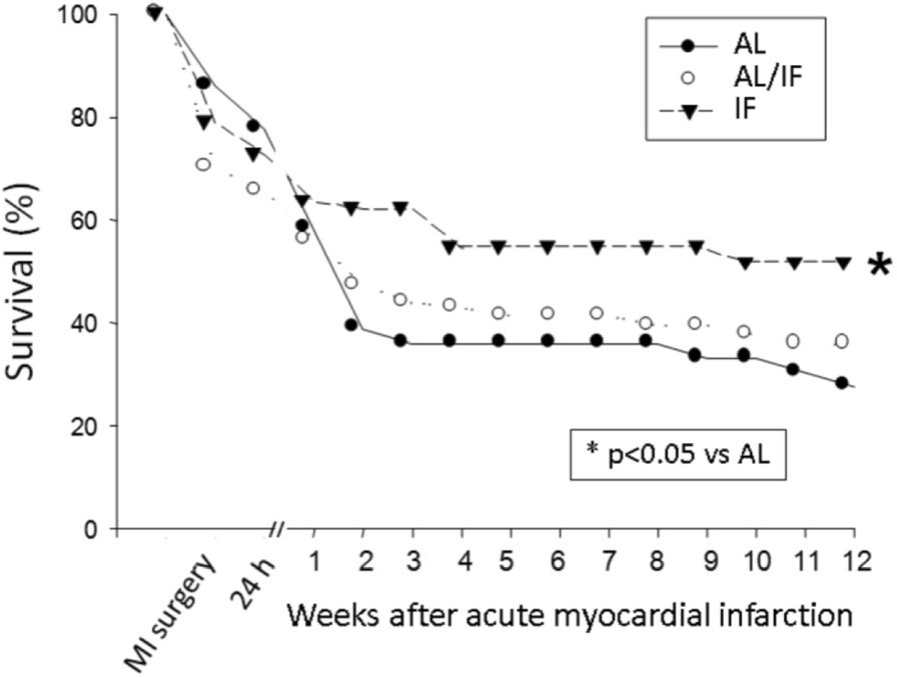
Kaplan-Meier curves for rat survival after myocardial infarction induction. AL: ad libitum fed; AL/IF: ad libitum before myocardial infarction (MI) and intermittently fasted after MI; IF: intermittently fasted before and after MI

Initial body weight (BW) did not differ between groups (Table 1). Before surgery, BW was lower in IF than AL and AL/IF groups. At the end of the experiment, AL/IF and IF had lower BW than AL. Perioperative mortality rate including the first 24 h after surgery did not change between AL (31.3%) and IF (27.3%) groups. At the end of the experiment, mortality rate was lower in IF than AL (Fig. 1).

**Table 1:** Body weight (g)

	AL	AL/IF	IF
Initial	283 ± 44	282 ± 39	281 ± 37
Before surgery	469 ± 45	459 ± 48	362 ± 33*^#^
Before euthanasia	481 ± 47	391 ± 41*	397 ± 45*

Data as mean ± standard deviation. *AL* ad libitum fed, *AL/IF* ad libitum before myocardial infarction (MI) and intermittently fasted after MI, *IF* intermittently fasted before and after MI. ANOVA and Tukey; * *p* < 0.05 vs AL; ^#^ *p* < 0.05 vs AL/IF

Before surgery, echocardiographic parameters of cardiac structure and function did not differ between AL and IF (data not shown). Echocardiographic data two weeks after MI induction are shown in Table 2. Isovolumic relaxation time in absolute and normalized to heart rate values were higher in IF than AL and AL/IF groups. Echocardiographic data 12 weeks after MI are shown in Table 3. LV diastolic posterior wall thickness was lower in AL/IF and IF than AL. E-wave deceleration time was lower in AL/IF than AL and IF. Myocardial infarction size did not differ between groups. The percentage of variation (Δ) of echocardiographic parameters between twelve and two weeks post-infarction was calculated as [(final value minus initial value)/initial value] X 100 (Table 4). Δ BW increased in all groups; the increase was higher in IF than AL/IF. Δ MI size decreased in all groups; the reduction in MI size was higher in IF than AL/IF.

**Table 2:** Echocardiographic evaluation two weeks after myocardial infarction

	AL (*n* = 35)	AL/IF (*n* = 39)	IF (*n* = 40)
BW (g)	439 ± 51	397 ± 44*	344 ± 23*^#^
HR (beats/min)	287 ± 27	280 ± 47	286 ± 34
LVDD (mm)	10.9 ± 0.87	10.9 ± 0.77	10.6 ± 0.99
LVSD (mm)	8.94 ± 1.16	8.99 ± 1.03	8.50 ± 1.37
LVDPWT (mm)	1.52 ± 0.15	1.51 ± 0.14	1.48 ± 0.14
LA (mm)	8.14 ± 0.86	8.14 ± 1.09	8.00 ± 1.23
E-wave (cm/s)	100 (93.5–118)	100 (91.0–110)	96.0 (81.0–110)
A-wave (cm/s)	20.0 (15.0–48.5)	19.0 (15.3–31.8)	19.0 (17.0–49.0)
E/A	5.50 (2.12–7.04)	5.79 (2.97–6.80)	5.17 (1.54–6.32)
IVRT (ms)	29.3 ± 4.06	30.8 ± 6.56	34.7 ± 5.90*^#^
IVRTn	64.2 ± 10.5	66.5 ± 14.9	75.4 ± 12.8*^#^
EDT (ms)	39.0 (30.8–44.3)	34.5 (27.0–39.0)	33.0 (30.0–39.0)
LV diastolic area (cm^2^)	0.91 ± 0.14	0.91 ± 0.15	0.88 ± 0.17
LV systolic área (cm^2^)	0.64 ± 0.14	0.64 ± 0.13	0.61 ± 0.15
Δ Area (%)	30.2 ± 7.77	30.8 ± 7.07	30.4 ± 9.10
MI size (% of total LV area)	50.0 ± 7.64	51.4 ± 6.66	52.3 ± 11.9

Data as mean ± standard deviation or median and 25th and 75th percentiles. *AL* ad libitum fed, *AL/IF* ad libitum before myocardial infarction (MI) and intermittently fasted after MI, *IF* intermittently fasted before and after MI. *BW* body weight, *HR* heart rate, *LVDD and LVSD* left ventricular (LV) diastolic and systolic diameters, respectively, *LVDPWT* LV diastolic posterior wall thickness, *LA* left atrial diameter, *E-wave and A-wave* early and late diastolic mitral inflow, respectively, *IVRT* isovolumic relaxation time, *IVRTn* IVRT normalized to heart rate, *EDT* E-wave deceleration time; Δ Area: fractional area change. ANOVA and Tukey or Kruskal-Wallis and Dunn; * *p* < 0.05 vs AL; # *p* < 0.05 vs AL/IF

**Table 3:** Echocardiographic evaluation twelve weeks after myocardial infarction

	AL (*n* = 26)	AL/IF (*n* = 28)	IF (*n* = 20)
BW (g)	479 ± 58	393 ± 41*	387 ± 28*
HR (beats/min)	283 ± 41	279 ± 26	261 ± 25*
LVDD (mm)	11.1 ± 0.98	10.7 ± 1.34	10.7 ± 0.62
LVSD (mm)	8.87 ± 1.32	8.66 ± 1.75	8.82 ± 1.13
LVDPWT (mm)	1.64 ± 0.14	1.48 ± 0.15*	1.48 ± 0.17*
LA (mm)	8.25 ± 1.48	7.92 ± 1.50	7.70 ± 1.17
E-wave (cm/s)	95.2 ± 28.0	88.9 ± 19.9	82.2 ± 21.9
A-wave (cm/s)	27.0 (19.0–43.0)	17.0 (14.5–38.5)	26.0 (15.0–46.0)
E/A	3.99 (1.37–5.46)	5.77 (1.68–6.73)	3.18 (1.48–6.63)
IVRT (ms)	34.8 ± 5.48	34.3 ± 7.00	33.3 ± 6.59
IVRTn	75.4 ± 12.6	73.7 ± 15.0	69.4 ± 13.7
EDT (ms)	36.0 (33.0–41.5)	30.0 (27.0–38.8)*	36.0 (33.0–42.0)^#^
LV diastolic area (cm^2^)	1.01 ± 0.18	0.94 ± 0.23	0.90 ± 0.18
LV systolic area (cm^2^)	0.68 ± 0.18	0.66 ± 0.21	0.60 ± 0.15
Δ Area (%)	33.0 ± 8.91	30.8 ± 9.92	33.5 ± 7.74
MI size (% of total LV area)	45.4 ± 8.67	48.4 ± 11.5	43.6 ± 9.90

Data as mean ± standard deviation or median and 25th and 75th percentiles. *AL* ad libitum fed, *AL/IF* ad libitum before myocardial infarction (MI) and intermittently fasted after MI. *IF* intermittently fasted before and after MI. *BW* body weight, *HR* heart rate, *LVDD and LVSD* left ventricular (LV) diastolic and systolic diameters, respectively, *LVDPWT* LV posterior wall thickness, *LA* left atrial diameter, *E-wave and A-wave* early and late diastolic mitral inflow, respectively, *IVRT* isovolumic relaxation time, *IVRTn* IVRT normalized to heart rate, *EDT*: E-wave deceleration time; Δ Area: fractional area change. ANOVA and Tukey or Kruskal-Wallis and Dunn; * *p* < 0.05 vs AL; # *p* < 0.05 vs AL/IF

**Table 4:** Percentage of variation (Δ) of echocardiographic parameters between twelve and two weeks post-infarction

	AL (*n* = 26)	AL/IF (*n* = 28)	IF (*n* = 20)
BW	7.86 (4.17–11.6)	4.35 (−2.61–6.90)	11.0 (9.57–13.0)^#^
HR	4.73 ± 15.4	4.25 ± 17.0	−7.91 ± 8.00*^#^
LVDD	7.61 ± 5.14	2.33 ± 8.45	2.85 ± 6.47
LVSD	6.02 ± 12.1	3.07 ± 13.2	6.75 ± 10.7
LA	8.40 ± 17.1	4.74 ± 9.87	−1.43 ± 15.0
E-wave	7.65 ± 33.9	−5.71 ± 17.2	− 14.4 ± 14.5*
A-wave	18.6 (−11.1–86.7)	−6.97 (−23.0–12.7)	−1.02 (−34.8–31.2)
E/A	0.23 (−47.5–15.1)	−8.14 (− 25.6–37.7)	−4.78 (− 34.2–29.2)
IVRT	14.6 ± 21.0	3.90 ± 22.9	− 2.96 ± 21.9
IVRTn	16.7 ± 21.1	5.21 ± 22.3	− 7.08 ± 20.7*
EDT	- 6.13 ± 20.7	− 11.1 ± 20.7	12.2 ± 30.6^#^
LV diastolic area	10.6 ± 12.0	8.89 ± 14.7	5.27 ± 18.5
LV systolic area	8.37 ± 22.9	12.1 ± 19.9	3.17 ± 19.0
Δ Area	9.13 ± 30.0	−3.06 ± 29.9	9.06 ± 35.0
MI size	−9.76 (−14.9- -2.49)	−4.13 (− 12.9–9.51)	− 13.2 (−23.7- -6.52)^#^

Data as mean ± standard deviation or median and 25th and 75th percentiles. AL: ad libitum fed; AL/IF: ad libitum before myocardial infarction (MI) and intermittently fasted after MI; IF: intermittently fasted before and after MI. Δ was calculated as [(final value minus initial value)/initial value] X 100. BW: body weight; HR: heart rate; LVDD and LVSD: left ventricular (LV) diastolic and systolic diameters, respectively; LA: left atrial diameter; E-wave and A-wave: early and late diastolic mitral inflow, respectively; IVRT: isovolumic relaxation time; IVRTn: IVRT normalized to heart rate; EDT: E-wave deceleration time; Δ Area: fractional area change. ANOVA and Tukey or Kruskal-Wallis and Dunn; * p < 0.05 vs AL; # *p* < 0.05 vs AL/IF

**Fig. 2:**
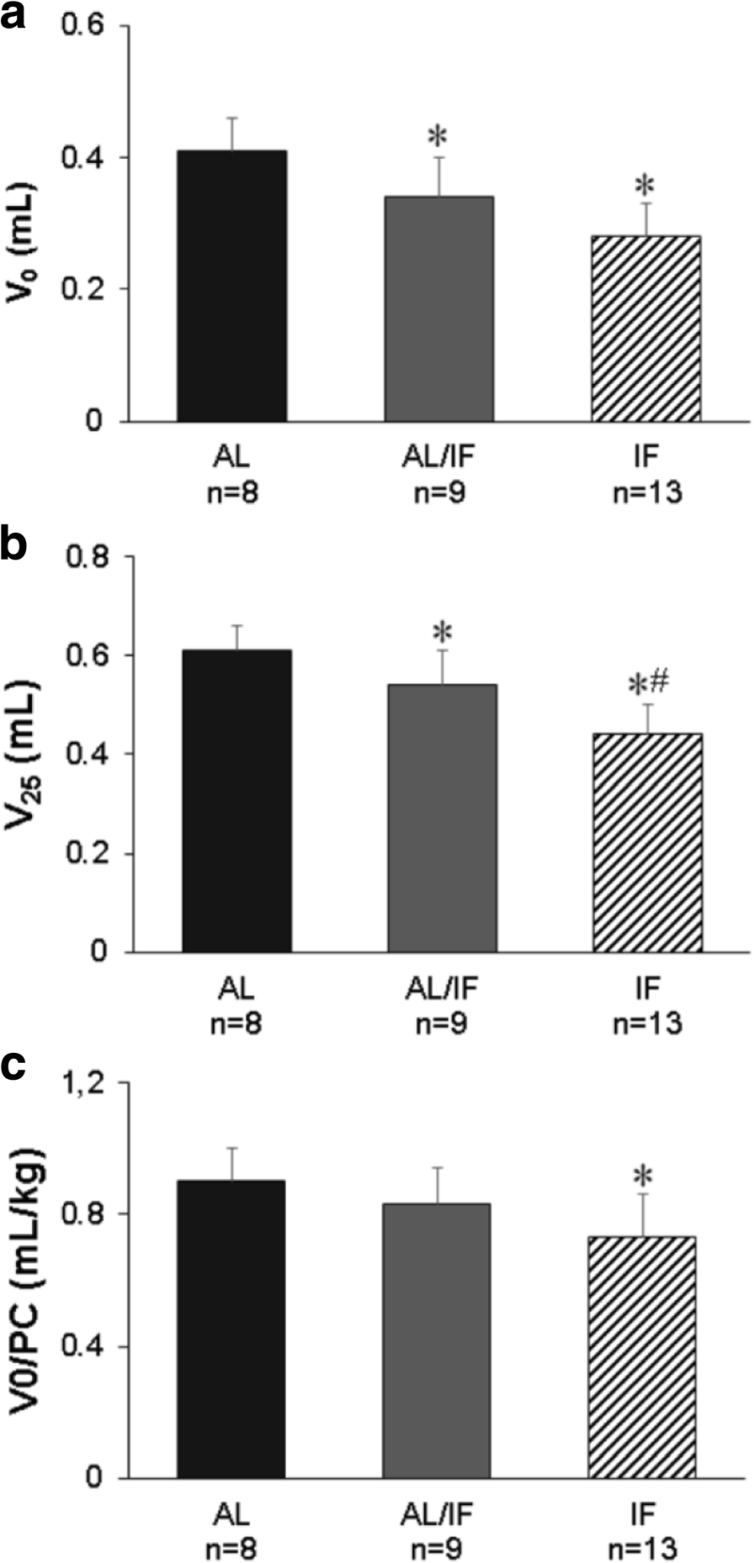
Left ventricular volume at zero (V_0_; a) and 25 mmHg (V_25_; b) end-diastolic pressure, and V_0_ normalized to body weight (V_0_/BW; c) 12 weeks after myocardial infarction (MI). AL: ad libitum fed; AL/IF: ad libitum fed before MI and intermittently fasted after MI; IF: intermittently fasted before and after MI. ANOVA and Tukey; *p < 0.05 vs AL; #*p* < 0.05 vs AL/IF

Isolated heart functional study data are presented in Table 5 and Fig. 2. V_0_ and V_25_ were lower in AL/IF and IF than AL and V_25_ was lower in IF than AL/IF; when normalized to BW, V_0_ was lower in IF than AL (Fig. 2). LV weight normalized to V_0_ was higher in IF than AL (Table 4).

**Table 5:** Isolated heart functional study

	AL (*n* = 8)	AL/IF (*n* = 9)	IF (*n* = 13)
BW (g)	458 ± 35	404 ± 39*	389 ± 32*
LVW (g)	1.05 ± 0.12	1.06 ± 0.21	0.99 ± 0.13
LVW/BW (g/kg)	2.29 ± 0.13	2.62 ± 0.40	2.54 ± 0.32
LVW/V_0_ (g/mL)	2.56 (2.18–2.90)	2.90 (2.54–3.92)	3.35 (3.11–3.75)*
DP_0_ (mmHg)	44.7 ± 16.9	30.8 ± 11.6	41.4 ± 16.7
DP_25_ (mmHg)	53.6 ± 22.3	50.0 ± 17.8	53.0 ± 26.5
DP_max_	58.3 ± 22.0	51.4 ± 18.0	56.3 ± 25.0
+dP/dt (mmHg/s)	1562 ± 590	1034 ± 385	1495 ± 643
Systolic stress_25_ (g/cm^2^)	80.8 ± 30.0	70.0 ± 31.0	65.0 ± 33.4
-dP/dt (mmHg/s)	1000 ± 340	562 ± 283	831 ± 341
Δ V_25_ (%)	49.0 ± 6.20	61.0 ± 21.5	57.0 ± 17.3
Strain 20 g/cm^2^ (%)	5.89 ± 0.92	7.50 ± 2.19	7.00 ± 1.88

Data as mean ± standard deviation or median and 25th and 75th percentiles. *AL* ad libitum fed, *AL/IF* ad libitum before myocardial infarction (MI) and intermittently fasted after MI, *IF* intermittently fasted before and after MI. *BW* body weight, *LVW* left ventricular (LV) weight, *DP*_*0*_ LV developed pressure at zero diastolic pressure, *DP*_*25*_ LV developed pressure at diastolic pressure of 25 mmHg, *DP*_*max*_ maximum developed pressure, *+dP/dt* maximum rate of pressure development; *Systolic stress*_*25*_ LV systolic stress at diastolic pressure of 25 mmHg, *−dP/dt* maximum rate of ventricular pressure decline, *Δ V*_*25*_ percentage of variation in LV volume required to increase diastolic pressure from 0 to 25 mmHg, *Strain 20 g/cm*^*2*^ percentage of myocardial strain caused by a diastolic stress of 20 g/cm^2^. ANOVA and Tukey or Kruskal-Wallis and Dunn; * *p* < 0.05 vs AL; # *p* < 0.05 vs AL/IF

Myocyte diameter was lower in AL/IF and IF than AL (AL 17.3 ± 1.70; AL/IF 15.1 ± 2.21; IF 13.4 ± 1.49 μm; *p* < 0.05 AL/IF and IF vs AL). Myocardial hydroxyproline concentration (AL 5.35 ± 2.39; AL/IF 8.16 ± 1.16; IF 7.39 ± 3.19 μg/mg myocardial tissue) and gene expression of ANP, Serca 2a, and α- and β-myosin heavy chain (Table 6) did not differ between groups.

**Table 6:** Gene expression

	AL (*n* = 5)	AL/IF (*n* = 6)	IF (n = 6)
ANP	1.00 ± 0.47	0.74 ± 0.16	0.58 ± 0.07
Serca 2a	1.00 ± 0.20	1.17 ± 0.15	1.09 ± 0.07
α-MHC	1.00 ± 0.17	1.01 ± 0.17	0.90 ± 0.10
β-MHC	1.00 ± 0.25	0.78 ± 0.07	0.72 ± 0.12

Data as mean ± standard deviation. *AL* ad libitum fed, *AL/IF* ad libitum before myocardial infarction (MI) and intermittently fasted after MI, *IF* intermittently fasted before and after MI. *ANP* atrial natriuretic peptide, *Serca 2a* sarcoplasmic reticulum calcium ATPase, *MHC* myosin heavy chain. ANOVA

## Discussion

The novel design of this study is the comparison of intermittent fasting initiated before and after myocardial infarction on rat cardiac remodeling.

Caloric restriction has long been shown to increase lifespan in rodents [34]. Moderate caloric restriction was associated with cardiac ageing delay, improved pressure overload-induced cardiac remodeling, and better myocardial ischemic tolerance [35–39]. On the other hand, severe caloric restriction impaired structural and functional parameters in spontaneously hypertensive rats [40]. Only more recently were cardiovascular parameters analyzed in rodents under intermittent fasting. Intermittent fasting in normal rats produced controversial results. Castello et al. [41] observed protection against age-induced inflammation, fibrosis, and oxidative stress in rat hearts. In contrast, increased fibrosis with diastolic dysfunction and diminished cardiac reserve were observed after six months of alternate-day fasting [42].

Body weight in intermittently fasted rats is usually lower than their ad libitum fed counterparts [13, 14]. In this study, AL/IF and IF groups had body weights approximately 18% lower than AL. However, body weight did not differ between rats that were subjected to intermittent fasting for 12 and 24 weeks showing that body weight loss following intermittent fasting is limited.

We have previously characterized myocardial infarction-induced cardiac remodeling in rats with large infarction size, usually characterized as an infarcted area greater than 40% of total LV area [15, 43, 44]. Six months after MI induction, rats with large MI present LV and left atrial dilation, LV diastolic posterior wall thickness increase, and systolic and diastolic dysfunction [15, 31, 43]. Feeding regimen in this study did not change MI size as all groups had large infarction size. This finding diverges from those by Ahmet et al. [13] who found smaller infarction size in intermittent fasted rats 24 h after MI induction. Our first evaluation of MI size was performed two weeks after MI. At this time, mortality was lower in IF than AL and AL/IF groups. Therefore, it is possible that rats with larger MI size in AL and AL/IF groups had already died before our first evaluation. Mortality throughout the 12 weeks post-infarction was lower in IF than AL; the AL/IF group had intermediate mortality and did not differ from both AL and IF groups.

Two weeks after MI induction, echocardiographic parameters did not differ between groups, except for a higher isovolumic relaxation time in the IF group. Twelve weeks after MI, LV diastolic posterior wall thickness was lower in AL/IF and IF than AL, showing that intermittent fasting attenuated myocardial hypertrophy. This finding was corroborated by lower myocyte diameter in both AL/IF and IF groups. When comparing changes in echocardiographic parameters between twelve and two weeks post-infarction, IF had a better remodeling than AL and AL/IF. This was characterized by a decrease in Δ E-wave and in Δ MI size and an increase in Δ E-wave deceleration time (EDT). As E/A ratio increases and EDT decreases in large infarctions [43], the changes in these parameters between both evaluations suggest a beneficial effect of intermittent fasting on diastolic function.

Results from Langendorff preparations confirmed a better effect of intermittent fasting when started before MI. V_0_ and V_25_ were lower in both intermittent fasted groups and V_25_ was lower in IF than AL/IF. These parameters indicate the LV volume at zero and 25 mmHg end-diastolic pressure, respectively. Furthermore, LV weight/V_0_ ratio was higher and V_0_/body weight ratio lower in IF than AL, suggesting a predominance of concentric over eccentric remodeling in the IF group. These data show that, despite the same MI size and therefore the same degree of ischemia-induced myocardial injury, intermittent fasting reduced LV cavity size, thus attenuating cardiac remodeling, and this attenuation was more intense in IF than AL/IF.

As potential mechanisms involved in cardiac remodeling attenuation, we evaluated myocardial expression of fetal genes and cardiac fibrosis. Interestingly, improvement in cardiac remodeling was not associated with changes in expression of the fetal genes α- and β-myosin heavy chain, atrial natriuretic peptide or Serca 2a. Reactivation of the fetal gene program is considered to be involved in adverse cardiac remodeling and the pathogenesis of heart failure [45]. Therefore, our results show that fetal gene program changes are not modulated by intermittent fasting. In the same way, myocardial hydroxyproline concentration, a marker of interstitial fibrosis, was not affected by dietary intervention. The effect of intermittent fasting on myocardial fibrosis is controversial, as both increased [42] and reduced [40] fibrosis was observed in healthy rats.

Attenuation of cardiac remodeling has previously been observed in rats subjected to intermittent fasting either before MI induction [13] or two weeks after MI [14]. As Ahmet et al. [13] reported, intermittent fasting reduced MI size therefore attenuating all aspects of cardiac remodeling. However, even in rats with similar MI sizes, intermittent fasting reduced myocyte apoptosis and neutrophil infiltration in the area at ischemia risk when compared to the ad libitum regimen [13].

Intermittent fasting initiated two weeks after MI accelerated pro-angiogenic and cell survive cascades [14]. To the best of our knowledge, this is the first study to show that the effects of intermittent fasting initiated before MI are more evident than when initiated after MI. Although both regimens decreased mortality, only when started before MI did intermittent fasting reach statistical significance. Both fasting regimens reduced myocyte hypertrophy and LV dilation. However, the IF group also had evidence of decreased MI size, concentric remodeling predominating over eccentric remodeling, and better diastolic function evolution than AL/IF rats. Additional studies are needed to clarify the mechanisms involved in the attenuation of myocardial infarction-induced cardiac remodeling.

## Conclusion

Intermittent fasting initiated before or after myocardial infarction reduces myocyte hypertrophy and left ventricular dilation in rats. Myocardial fibrosis and fetal gene expression are not modulated by the feeding regimens. Benefit is more evident when intermittent fasting is initiated before than after myocardial infarction.

## Data Availability

The datasets used and/or analysed during the current study available from the corresponding author on request.

## Abbreviations

+dP/dt: Maximum rate of pressure development
AL: Ad libitum
ANOVA: Analysis of variance
ANP: Atrial natriuretic peptide
-dP/dt: Maximum rate of ventricular pressure decline
E and A waves: Early and late, respectively, diastolic mitral inflow velocities
EDT: E-wave deceleration time
IF: Intermittent fasting
IM: Myocardial infarction
IVRT: Isovolumetric relaxation time
IVRTn: IVRT normalized to heart rate
LA: Left atrial diameter
LV: Left ventricular
LVDD and LVSD: LV diastolic and systolic dimensions, respectively
LVDPWT: LV diastolic posterior wall thickness
LVV: LV volume
LVW: LV weight
V_0_: LV volume at zero end-diastolic pressure
V_25_: LV volume at 25 mmHg end-diastolic pressure
Δ area: Fractional area change
Δ V_25_: Percentage of variation in LV volume required to increase diastolic pressure from 0 to 25 mmHg
Δ: Percentage of variation.
